# Declining coastal piscivore populations in the Baltic Sea: Where and when do sticklebacks matter?

**DOI:** 10.1007/s13280-015-0665-5

**Published:** 2015-05-28

**Authors:** Pär Byström, Ulf Bergström, Alexander Hjälten, Sofie Ståhl, David Jonsson, Jens Olsson

**Affiliations:** Department of Ecology and Environmental Science, Umeå University, 901 87 Umeå, Sweden; Department of Aquatic Resources, Institute of Coastal Research, Swedish University of Agricultural Sciences, Skolgatan 6, 742 42 Öregrund, Sweden; County Administrative Board of Västernorrland, 871 86 Härnösand, Sweden

**Keywords:** Intraguild predation, Ecosystem coupling, Recruitment, Coastal piscivores, Three-spined stickleback, Alternative stable states

## Abstract

Intraguild predation interactions make fish communities prone to exhibit alternative stable states with either piscivore or prey fish dominance. In the Baltic Sea, local declines of coastal piscivores like perch (*Perca fluviatilis*) have been observed to coincide with high densities of sticklebacks (*Gasterosteus aculeatus*). Mechanisms behind this shift between piscivore and stickleback dominance were studied both experimentally and in field. Results showed that predation by sticklebacks has a strong negative effect on perch larvae survival, but this effect rapidly decreases with increasing perch size, likely due to gape limitations and digestion constraints in sticklebacks. Large spatial and temporal variations in patterns of stickleback migration into perch spawning sites were observed. Whether or not high density of sticklebacks will cause declines in coastal piscivore populations is suggested to depend on the availability of spawning sites in which sticklebacks do not migrate into or arrive late in the reproduction season of coastal piscivores.

**Electronic supplementary material:**

The online version of this article (doi:10.1007/s13280-015-0665-5) contains supplementary material, which is available to authorized users.

## Introduction

Life history omnivory (shifts in resource use from invertebrates to fish over ontogeny) is common in fish and may involve both habitat shifts and interactions where species both prey on and compete with each other, i.e., intraguild predation (IGP) (Werner and Gilliam 1984; Irigoien and de Roos 2011). IGP in fish communities is strongly dependent on size relationships between interacting species and includes interactions where juvenile predators compete with their future prey for shared resources to interactions where prey feed on juvenile predators, i.e., reciprocal IGP (Persson et al. 2007a; Fauchald 2010; van der Hammen et al. 2010; Hin et al. 2011). In the former case, competition from prey for shared resources may result in a juvenile competitive bottleneck in the predator which limits recruitment to the adult predator stage (Werner and Gilliam 1984; Byström et al. 1998). In the latter case, prey may impose high mortality on juvenile predators through predation and thereby reduce the recruitment of predators (Fauchald 2010). Based on either or both of above mechanisms, Walters and Kitchell (2001) suggested the presence of two alternative stable community states (ASS), a high predator density state where predators control the prey population such that they increase the growth and survival of their own offspring, or a high prey population density state were prey limit predator population growth and density. The potential for both competition and reciprocal predation control in IGP systems is especially challenging in a management context. This is due to that these systems may initially respond to exploitation of predators or gradual environmental change similar to traditional linear food chains until reaching thresholds where sudden regime shifts occur (Hin et al. 2011; Gårdmark et al. 2015). Thus, in a high prey population density state, a recovery of a predator population may be hampered by the strong negative effects from the prey population on predator recruitment (Walters and Kitchell 2001; Fauchald 2010; Gårdmark et al. 2015).

Ecosystems or habitats are in many cases coupled through fluxes of organisms (Massol et al. 2011) and in spatially linked systems with IGP, the likelihood for ASS has been shown to depend on the degree of spatial separation (van der Hammen et al. 2010) or on the relative productivity of the coupled systems (Schreiber and Rudolf 2008). In marine ecosystems, migrating fish that over their life cycle use different habitats for growth and reproduction, link processes in offshore, and coastal and/or freshwater ecosystems (Schindler et al. 2005; Eriksson et al. 2011). Moreover, ecosystem-specific anthropogenic impacts like fishing or eutrophication may cause strong effects in the adjacent ecosystems through changes in densities of migrating fish (Eriksson et al. 2011; Casini et al. 2012). Consequently, in offshore–coastal IGP systems, the potential for both cross-ecosystem migration of fish and variation in habitat-specific productivity could cause ASS in either of the systems.

Eutrophication and depletion of large piscivores have resulted in dramatic changes in the Baltic Sea ecosystem (Österblom et al. 2007; Möllmann et al. 2009). The collapse of cod (*Gadus morhua*) has resulted in predation release of small planktivores, and a planktivore dominated offshore ecosystem (Casini et al. 2009; Eriksson et al. 2011). Along with this shift in the offshore ecosystem, local declines in the Baltic Proper have been observed of the coastal keystone piscivorous fish species: perch (*Perca fluviatilis*) and pike (*Esox lucius*) (Ljunggren et al. 2010; Eriksson et al. 2011). This decline has coincided with increasing densities of sticklebacks which migrate into coastal areas for reproduction (Nilsson et al. 2004; Ljunggren et al. 2010; Eriksson et al. 2011; Appelberg et al. 2013). This shift from piscivore dominance to a coastal fish community dominated by sticklebacks has been suggested to be due to competition and/or predation from increasing densities of three-spined sticklebacks on young piscivores. However, evidence for this is circumstantial and based mainly on negative correlations between stickleback and young-of-the-year (YOY) perch abundance in field data (Nilsson 2006; Ljunggren et al. 2010; Eriksson et al. 2011). Decreased top–down control of sticklebacks from declining coastal piscivore populations has also been suggested to increase eutrophication symptoms, i.e., algal blooms in shallow bays, as sticklebacks feed on grazers which in turn controls algal biomass (Eriksson et al. 2011; Sieben et al. 2011). Hence, in addition to their importance for recreational and commercial small-scale fisheries, coastal piscivore populations provide important regulating ecosystem services in being key species for the function and structure of coastal food webs (HELCOM 2007; Bergström et al. 2013).

The cascading effects of high densities of sticklebacks on lower trophic levels in the Baltic Sea coastal ecosystem are well known (Eriksson et al. 2011, and references therein), but whether or not sticklebacks have negative impacts on coastal piscivore population densities and what mechanisms may be operating is still an open question. The aim here was to study if sticklebacks can have a negative impact on the survival of perch recruits, and under which conditions this may be important for recruitment success of coastal piscivores. More specifically, we studied what sizes of YOY perch are vulnerable to stickleback predation in laboratory and estimated mortality rates of differently sized YOY perch in the absence or presence of sticklebacks in a large-scale pond experiment. The results from these studies were then contrasted to field data on temporal variation in stickleback migration into piscivore spawning sites and mortality patterns of YOY perch in relation to stickleback densities. Finally, in a coastal area with high densities of stickleback we estimated (a) the number of perch migrating from the sea up to a small freshwater lake for spawning, (b) the YOY perch densities in that lake, and (c) the proportion of perch that have been recruited from freshwater systems in that area.

## Materials and methods

Detailed descriptions of methods for all approaches can be found in Supplementary Material S1.

### Laboratory experiment and gape size limitation

In order to estimate gape limitations of three-spined sticklebacks when feeding on differently sized YOY perch, we measured stickleback gape sizes and conducted feeding experiments in aquarium using different size combinations of sticklebacks as predators and perch as prey. Gape sizes of a random sample of sticklebacks were measured to the nearest 0.1 mm as the distance between the upper and lower jaw at a gape angle of 90°, using a stereo microscope. To estimate theoretical maximum length of a YOY perch that sticklebacks can consume, we combined our estimate of stickleback gape size relationship with published relationships between YOY perch body height and length. For the feeding experiments, we used three size classes of stickleback: small (30–40 mm), medium (45–55 mm), and large (65–75 mm) placed individually in aquarium and six size classes of YOY perch as prey (11, 14, 19, 21, 25, and 31 mm, total length). Prior to experiments, sticklebacks were starved for 24 h, and thereafter either ten YOY perch (size classes 11 and 14 mm) or five YOY perch (size classes 19, 21, 25, and 31 mm) were introduced to each aquarium. Number of consumed perch was recorded after 4 h.

### Pond experiment

We conducted a large-scale pond experiment to examine whether or not survival of YOY perch is dependent on their size when three-spined sticklebacks migrate into perch spawning sites. The experiment was conducted in two ponds (32 × 10.8 m) with eight enclosures each (size 4 × 10.8 m, mean depth 0.90 m). We used a design with four treatments with temporal variation in stickleback presence and one control (no sticklebacks) replicated 3 times each. Three hundred (6.9 individuals m^−2^) first feeding perch larvae (7.2 ± 0.26 mm, mean ± 1 SD) were introduced at 28th of May into each of the enclosures. Thereafter, six (0.14 individuals m^−2^) adult three-spined sticklebacks (62.5 ± 4.7 mm) were introduced either 1, 8, 17, or 24 days after the introduction of perch larvae. The stickleback density chosen is low compared to natural densities where up to 30 individuals m^−2^ could be found in some coastal areas (Eriksson et al. 2011). In each treatment, perch and sticklebacks were sampled 18–19 days after introduction of sticklebacks, i.e., day 19, 27, 35, and 43. Fish were sampled in each enclosure with a fine mesh seine net. Zooplankton densities were sampled with a 100-µm mesh net (diameter 250 mm) drawn 3.5 m horizontally at a depth of 0.1 m in the deepest part of the enclosures at the introduction of perch larvae and at the day prior to termination of each treatment

### Field studies

#### Densities of perch larvae at coastal spawning sites

**Fig. 1:**
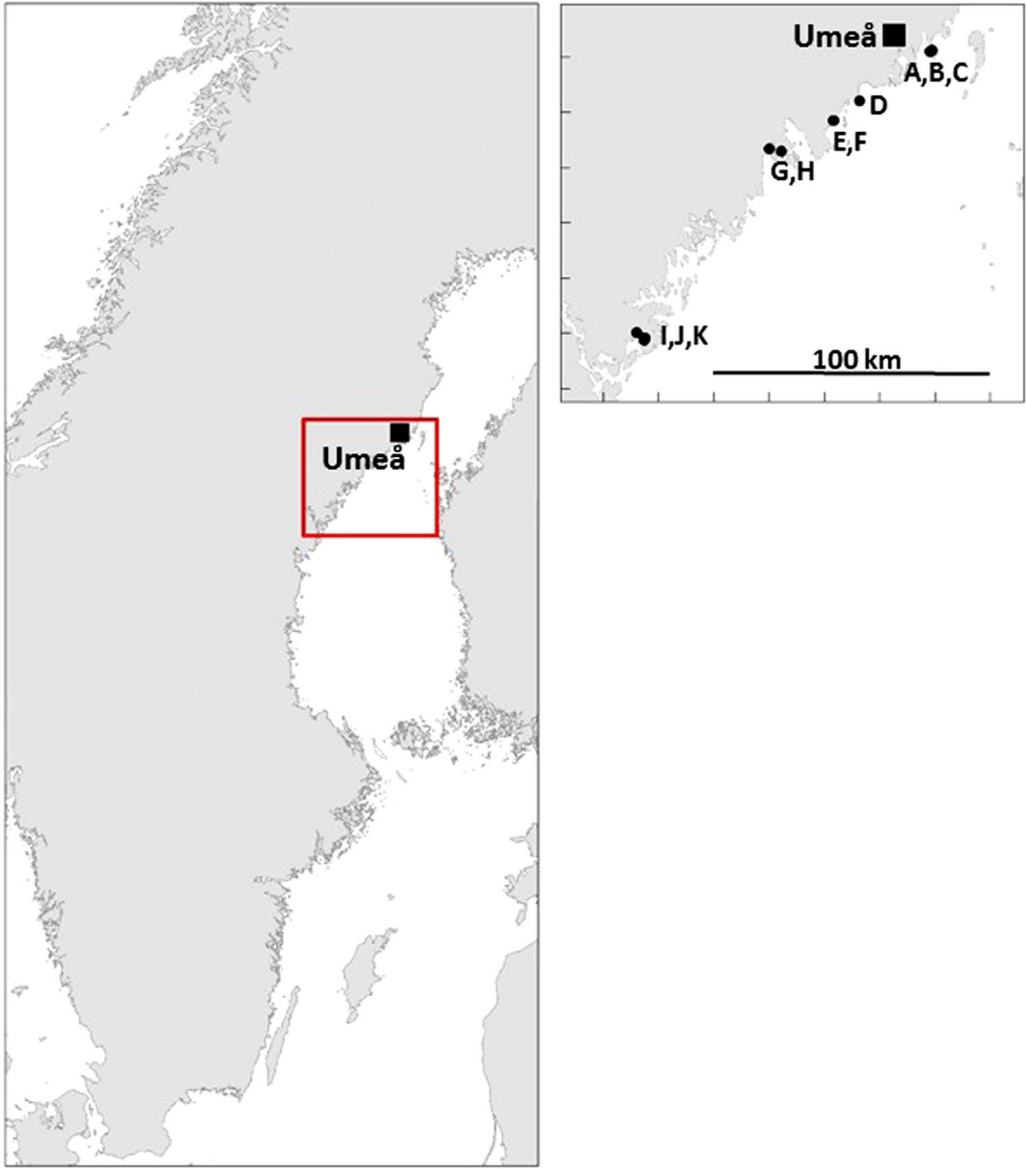
Studied spawning sites of perch in the Bothnian Sea. For site names and coordinates see Tables 1 and S1

In order to study relationships between changes in perch larvae and three-spined stickleback density over time, we sampled perch spawning sites along the Bothnian Sea coast in the years 2011 and 2012 (Fig. 1; Tables 1, S1) for perch larval densities and stickleback abundances. Larval perch were sampled weekly or every second week, approximately from hatching (Table 1) and five weeks onwards with a bongo-trawl. Trawling was made during day-time, and at least four stations were sampled in each bay and date. All captured larvae were counted, and individual subsamples were preserved in Lugol’s solution for later length measurement in the laboratory (total length, to the nearest 0.1 mm). Concomitant with the trawling, 16 to 22 Ella traps (www.ellafishing.com) were set over night approximately 10 m apart along the shoreline at a depth of 1–2 m to obtain a relative measure of stickleback abundance in each bay. Captured sticklebacks were counted and thereafter released back to the bay after a subsample of sticklebacks was collected and frozen for later diet analyses. Zooplankton abundance was sampled at three pelagic stations in each bay using a 100-µm mesh net (diameter 250 mm). Samples were preserved in Lugol’s solution. In this study, we report data on YOY perch abundances and stickleback catches from only two of the sampling sites, Yttre Spelgrundet and Västra Stadsviken, which were sampled in both 2011 and 2012 as the complete data set is used in another article (Byström and Wennhage, unpubl.), whereas we use the whole dataset to study variation in stickleback catches at perch the spawning sites.

**Table 1:** Hatching date for perch and mean CPUE (# trap^−1^ ± 1 SD) of adult sticklebacks at hatching of perch and at one to three weeks after hatching (# days given in brackets) at ten different perch spawning sites in the Bothnian Sea. *Based on hatching in Västra Stadsviken. **No larvae found and hatching date assumed to be the same as in Häggvik. n.a. data not available

Site	Year	Hatching date (mmdd)	CPUE at hatching	CPUE after hatching (# days)
(A) Laxögern	2011	0518	0	5.2 ± 0.9 (21)
(B) Yttre Spelgrundet	2011	0518	0	307 ± 90 (21)
	2012	0529	0	11 ± 9 (17)
(C) Inre Spelgrundet	2012	0529	0	15 ± 25 (17)
(D) Boviken	2011	0527	n.a.	767 ± 497 (7)*
(E) Västra Stadsviken	2011	0527	3.7 ± 1.4	197 ± 50 (7)
	2012	0524	1.6 ± 2.6	38 ± 50 (8)
(F) Östra Stadsviken	2012	0524	0.05 ± 0.1	13.2 ± 9.2 (14)
(G) Tennavan	2011	0517	0	0.1 ± 0.07 (21)
(H) Inneravan	2011	0517	0.1 ± 0.07	0.1 ± 0.07 (21)
(I) Sörleviken (Gaviksfjärden)	2011	0601	391 ± 525**	n.a.
(J) Häggvik (Gaviksfjärden)	2011	0601	51 ± 22	151 ± 52 (8)

#### Case study

Results from coastal survey gillnet monitoring programs show that three-spined stickleback abundance in the large coastal bay Gaviksfjärden (includes both sub-bays Häggvik and Sörleviken (Tables 1, S1; Fig. 1) has increased substantially from year 2004 to 2012 (Appelberg et al. 2013; Lingman 2013). Despite high densities of sticklebacks, the suggested negative effects of sticklebacks on perch populations have not been observed in Gaviksfjärden (Lingman 2013, Olsson et al. unpublished). In Gaviksfjärden, there are at least two freshwater outlets that connect the coast with closely situated freshwater lakes. To investigate the importance of freshwater lakes as spawning sites for perch in Gaviksfjärden, we estimated the number of upstream migrating perch to one of these the lakes between 2nd of May and 29th of May in spring 2013 with a fish counter (Vaki river fish counter). Bongo trawling was carried out during two occasions (29th of May and 6th of June) to estimate perch densities, and six Ella traps were set over night on the 29th of May and 6th of June in the lake to assess whether or not stickleback was present or migrated up to the lake. In addition, the contribution to the resident perch population in Gaviksfjärden of freshwater recruited perch was assessed using otolith micro-chemistry analysis of strontium (Sr) and calcium (Ca) concentrations on adult perch captured at the coast (Wastie 2013).

## Results

### Gape limitation

**Fig. 2:**
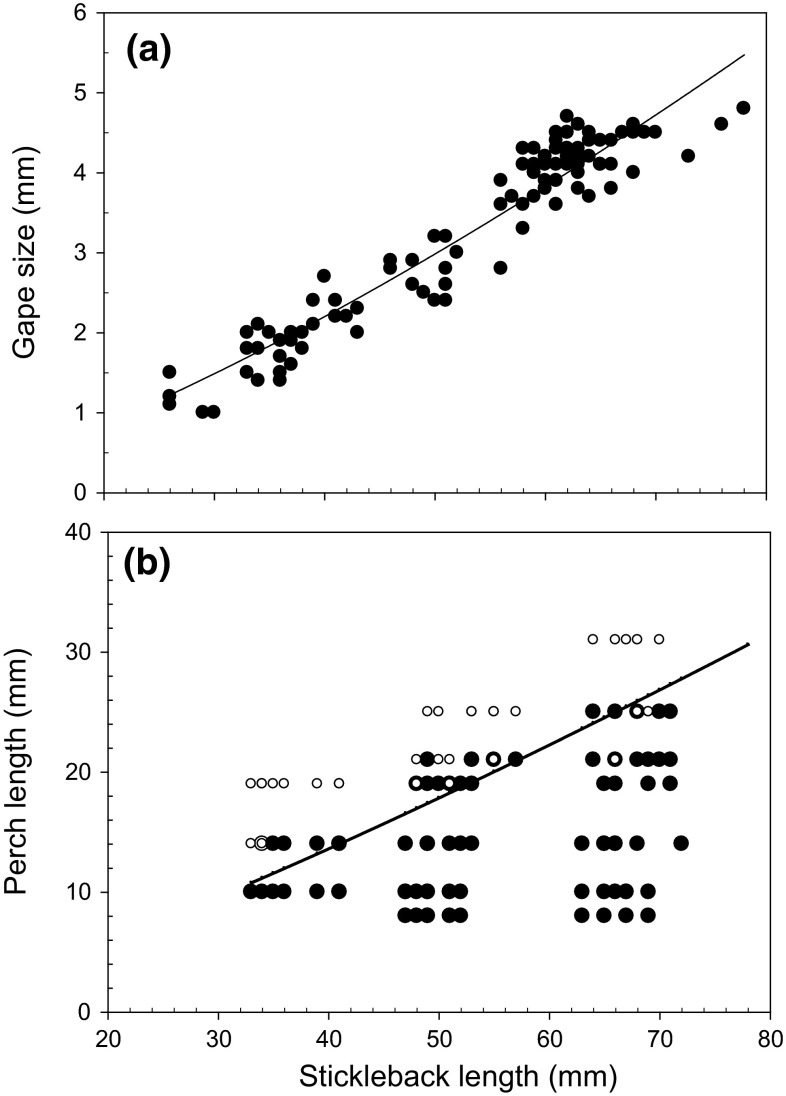
**a** The relationship between stickleback body length and gape size. **b** The relationship (*filled line*) between predicted maximum perch size (*length*) that differently sized (*length*) sticklebacks are able to consume (*line*). *Filled circles* represent perch larvae consumed by sticklebacks and *open circles* represent sizes of perch that differently sized stickleback was unable to consume. Overlapping data is represented by a *small white circle* with a thick black edge

Stickleback gape size increased monotonically with length (*L*) according to Gape size = 0.01 × *L*^1.457^ (*r*^2^ = 0.92, *P* < 0.001, Fig. 2a). Combining the gape size and perch body height relationships renders that the predicted maximum perch length (*P*_L_) that a stickleback can consume increases with stickleback length (*S*_L_) according to *P*_L_ = 0.515 × *L*^1.214^. According to the experiment, sizes of YOY perch that sticklebacks could consume were slightly lower than predicted above. The maximum size of YOY perch that an adult stickleback 60–70 mm in length can consume varied between 20 and 25 mm (Fig. 2b).

### Pond experiment

**Fig. 3:**
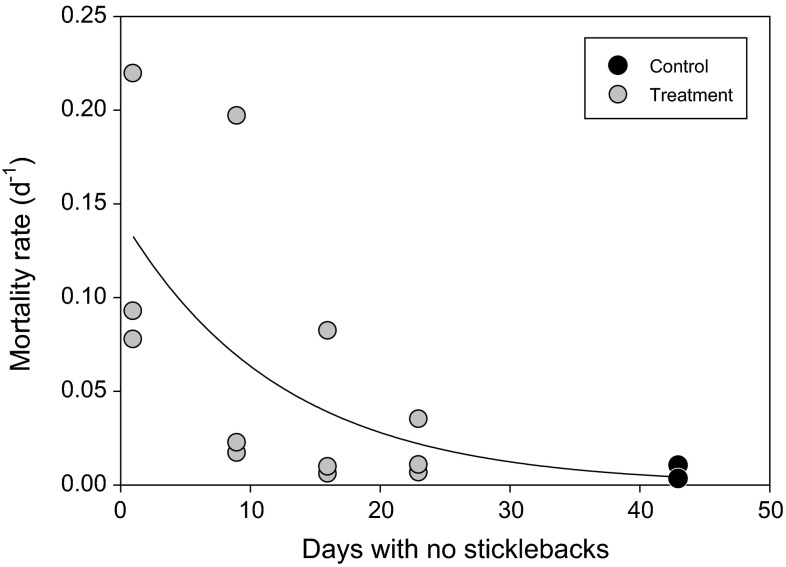
Mortality rates in treatments and controls of YOY perch as a function of number of days after hatching before sticklebacks were introduced into the enclosures. Note that rates are estimated based on the number of days each treatment and control lasted before sampling of surviving perch, i.e., 18, 27, 34, 42, and 43 days, respectively

Survival of perch was substantially higher (72.7 ± 13.1 %) in the absence of sticklebacks (controls) compared to the survival of perch in enclosures where sticklebacks were introduced at day one (11.3 ± 9.6 %; *t* test, *t* = 6.56, *P* = 0.002; see also Fig. 3 for mortality rates). Mortality rates of perch larvae decreased with the number of days after hatching before sticklebacks were introduced (non-linear regression, *r*^2^ = 0.44, *P* = 0.06; Fig. 3). Zooplankton biomass was not negatively affected in the presence of sticklebacks and zooplankton even increased over time when sticklebacks were introduced at day one (Repeated measure ANOVA: Stickleback effect *F*_1,4_ = 3.84–0.03, *P* = 0.12–0.88; Stickleback effect × time *F*_1,4_ = 9.18–0.21, *P* = 0.04–0. 44) (Fig. S1).

### Field studies

#### Spawning sites at the coast

**Fig. 4:**
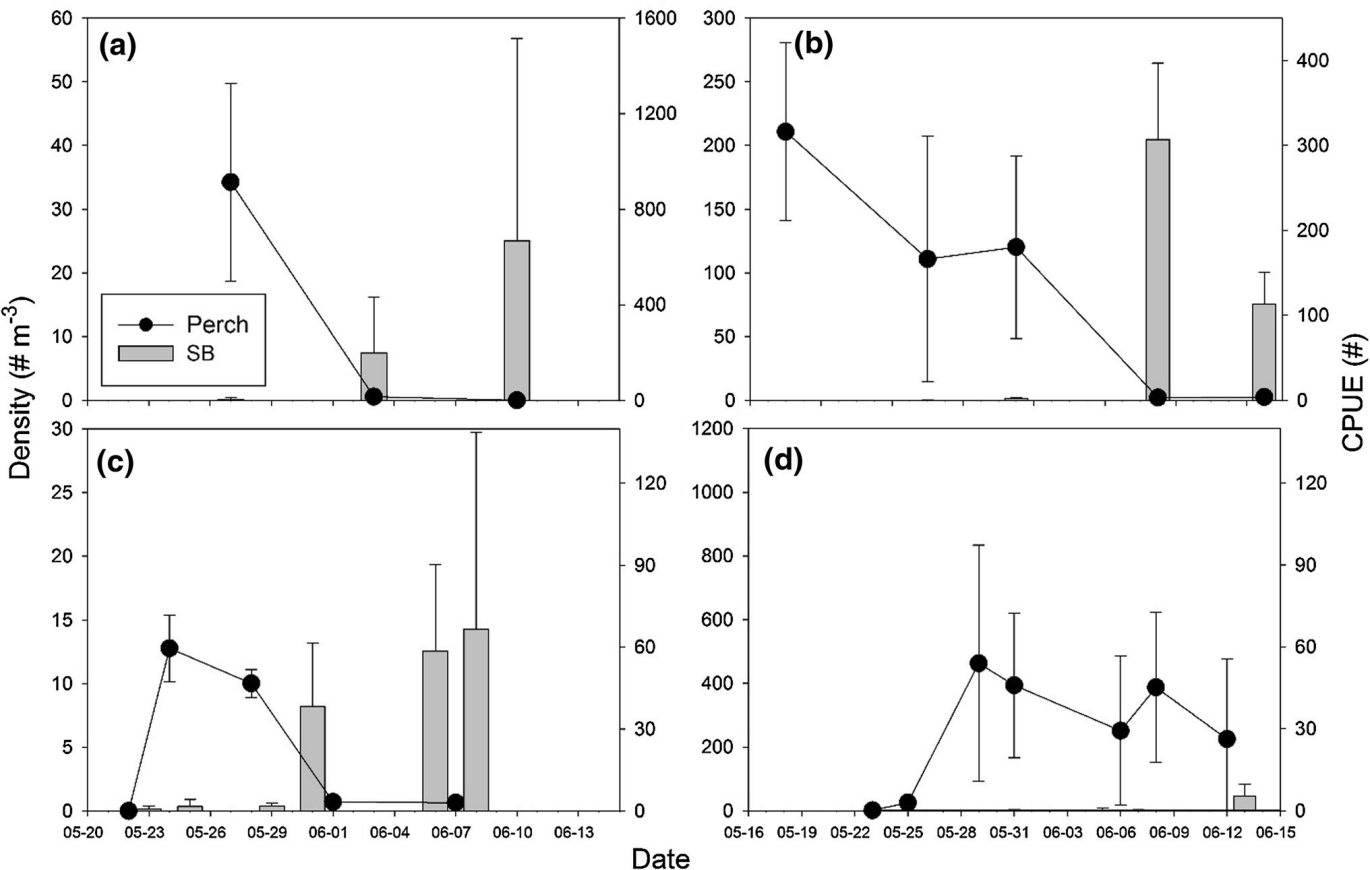
Densities of perch larvae (# m^−3^ ± 1 SD) and CPUE (# trap^−1^ ± 1 SD) of sticklebacks in Västra Stadsviken 2011 (**a**) and 2012 (**c**) and in Yttre Spelgrundet in 2011 (**b**) and 2012 (**d**). *Lack of bars* at trawling occasion indicates zero catches of sticklebacks

Densities of perch larvae varied little between trawling occasions up until an increase in Catch-per-Unit-Effort (CPUE) of sticklebacks took place, when a strong decrease in perch larval density was observed (Fig. 4a–d). Zooplankton biomasses (data not shown) did not decrease between the trawling occasions when these declines of perch larvae took place (paired *t* test, *t* = 1.04, *P* = 0.41). Stickleback densities at, and up to 21 days after, hatching of perch larvae varied strongly between spawning sites along the coast of the Bothnian Sea. Four out of ten sites had CPUE of stickleback above 30 individuals trap^−1^ at or within eight days from hatching, while others had almost none (Table 1).

#### Case study

In total 194 (29.1 ± 5.7 cm, mean total length ± 1 SD), perch were recorded migrating from the coastal Gaviksfjärden towards the lake from the 2nd to the 29th May, with a peak in migration around the 6–8th of May. Estimated densities of perch larvae in the lake were 177 ± 104 and 189 ± 72 individuals m^−3^ on the 29th of May and at the 6th of June, respectively. No sticklebacks were captured in the lake at either of the two sampling dates. All of the perch individuals from the coastal area were according to the Sr:Ca ratio in the core of their otoliths determined to have been recruited from freshwater systems.

## Discussion

### Effects of sticklebacks on perch survival

Both our experimental results and field data support the suggested negative impact of sticklebacks on coastal piscivore recruitment (Nilsson et al. 2004; Nilsson 2006; Ljunggren et al. 2010; Eriksson et al. 2011). Although we have only studied the performance of the smallest stages of YOY perch (up to 5–6 weeks from hatching), the strong negative effects on perch larval survival suggest that the negative effects of sticklebacks most likely operate on the earliest life stages. Competition for zooplankton from sticklebacks has previously been suggested to be the main reason for observed patterns of low recruitment in coastal piscivores (Ljunggren et al. 2010). In this study, we could not detect any negative effects of sticklebacks on zooplankton abundance, while strong decreases in perch larval abundances in the presence of sticklebacks were evident both in field and in the pond experiment. This suggests that predation from sticklebacks was the main mechanism behind the observed mortality patterns in larval perch. Correspondingly, stomach analyses have revealed that sticklebacks feed on both pike eggs (Nilsson 2006) and larval perch at spawning sites in the Baltic Sea (Byström and Wennhage, unpubl.). Similar patterns have also been found in lakes where perch larval survival has been shown to depend mainly on predator densities and not on density-dependent effects of planktivores on zooplankton densities (Persson et al. 2007a). The lack of resource-dependent effects on larval survival can in turn be related to general size scaling relationships between search and digestion capacities, which suggests that larval stages are rarely strongly resource limited (Persson et al. 2000). Based on above we therefore suggest that predation from sticklebacks alone can explain the observed strong negative effects from high densities of sticklebacks on perch larval survival.

As small predators in general (Fuiman 1994; Lundvall et al. 1999), sticklebacks become strongly gape limited if their prey grow in size, and our data show that adult stickleback can consume YOY perch up to a length of 20–25 mm. Increased reactive distance and swimming speed with larval development and size both improve escape capacities from predators (Fuiman 1994; Lundvall et al. 1999), and consumption rates of small predators are also constrained by low digestion capacities (Wootton et al. 1980; Persson et al. 2000). Hence, numerical effects on prey by small-sized predators like sticklebacks decrease strongly with prey size. For example, based only on size-dependent daily consumption capacities of sticklebacks, a 2.5-g stickleback (65–70 mm) can daily consume approximately 250–300 perch larvae with a length of 6.5 mm but only 10–12 larvae with a length of 15 mm (Wootton et al. 1980). Correspondingly, the pond experiment mortality rates of YOY perch decreased with increasing size when sticklebacks were introduced, and in our field study, the size of perch larvae was small (6–9 mm, Byström; unpubl.) at the time when strong decreases in their densities were observed. Thus, we suggest that stickleback predation can have strong negative effects on YOY perch recruitment, although this effect is expected to be strongest at the earliest larval stages and decline relatively rapidly as YOY perch increase in size.

### Spatial and temporal effects of sticklebacks on coastal piscivore recruitment and population densities in the Baltic Sea

Sticklebacks in the Baltic Sea link offshore and coastal ecosystems through their spring spawning migrations to the same shallow coastal bays as coastal piscivores may use for their reproduction (Nilsson et al. 2004; Ljunggren et al. 2010). The strong size dependence in the predation effects of sticklebacks on YOY piscivore survival suggests that temporal and spatial migration patterns of sticklebacks into piscivore spawning sites may have major effects on local recruitment success of coastal piscivores. Earlier studies have shown that the recruitment success of perch and pike, measured as densities of YOY fish in July–September, is close to binary with either no or low recruitment at high densities of YOY sticklebacks and high recruitment at low densities of YOY sticklebacks (Nilsson et al. 2004; Appelberg M. et al. 2013). Our data on the temporal variation in stickleback migration into spawning sites show large between-site variation, with very high densities of adult sticklebacks already at hatching of perch larvae at some sites to almost no sticklebacks present at other sites. Hence, spatiotemporal variation in stickleback migration into piscivore spawning sites combined with strong size dependency in predation mortality can be suggested as a mechanism explaining the almost dichotomous site-specific patterns in recruitment success of YOY piscivores along the Baltic Sea coast.

Distance to spawning sites, migration barriers, and climate-dependent onset of migration are factors that have shown to determine timing of arrival and accessibility of fish to spawning sites (Eriksson and Müller 1982; McCleave et al. 1984; Candolin and Voigt 2003). High stickleback densities and low piscivore recruitment have generally been observed in piscivore spawning areas closer to the open sea, from where sticklebacks migrate to shallow coastal spawning areas, while the opposite pattern is seen in inner archipelago areas (Ljunggren et al. 2010). Correspondingly, the distances to the open sea for the two most sheltered sites in this study Tennavan and Inneravan are 4 and 8 km, respectively, including narrow channels and outer bays to pass before entering the spawning sites. In these bays, no or very few sticklebacks were found. In contrast, Blåviken and Yttre Stadsfjärden have a distance to the open sea of 1–1.4 km, respectively, with no migration barriers, and the densities of sticklebacks were high around the time of hatching of perch larvae. Although sticklebacks in the Baltic Sea also may migrate up in freshwater systems, this migration seems to be restricted to estuaries or to the lowest parts of rivers (Eriksson and Müller 1982; Schaarschmidt and Jurss 2003). Water speed and migration barriers are likely to constrain upstream migration of sticklebacks to a substantially higher degree than the much larger perch which may migrate longer distances and up to coastal lakes for spawning (Eriksson and Müller 1982; Videler 1993). The use of freshwater habitats has been related to the benefits of relatively higher temperatures compared to coastal sites, in turn stimulating earlier hatching of larvae and onset of food production or to a response to low tolerance to salinity (Engstedt et al. 2010; Eriksson and Müller 1982; Tibblin et al. 2012). Our case study from Gaviksfjärden provides support for an additional benefit for perch of using freshwater habitats and sheltered bays in the inner archipelago for spawning, i.e., reduced predation from sticklebacks. In addition to the spatiotemporal patterns seen in the coastal bays, we found that perch migrated up to a coastal lake and that the densities of perch larvae in the lake were comparable to the densities in the studied coastal spawning sites. Taken together with the fact that all analyzed adult perch from this coastal site had been recruited in freshwater systems, these results suggest that the rather stable perch population over time in spite of high stickleback densities in Gaviksfjärden (Olsson et al. unpubl.; Lingman 2013) may be explained by the spatial segregation between the two species when perch larvae are most vulnerable to stickleback predation. Correspondingly, in the southern part of the Baltic Sea, in areas with strong declines in coastal piscivores and high densities of sticklebacks (Nilsson et al. 2004; Ljunggren et al. 2010; Eriksson et al. 2011) parts of the remaining Northern pike populations have been suggested to be recruited from freshwater systems (Engstedt et al. 2010).

In coastal areas with high densities of sticklebacks or other gape-limited predators, the size at which piscivore larvae and their predators meet likely has strong effects on their survival. Larvae hatching in coastal streams and rivers likely drifts passively out at hatching to the coast due to their low swimming capacities, whereas larvae hatching in lakes or flooded wetlands is more likely to stay and migrate out to the coast later and at a larger size (Eriksson and Müller 1982; Nilsson et al. 2014). Availability of suitable nursery habitats in the Baltic Sea has been suggested to strongly influence coastal piscivore population densities (Sundblad et al. 2014), and home ranges of coastal piscivores are typically less than 100 km (Saulamo and Neuman 2002; Laikre et al. 2005; Olsson et al. 2011), further indicating that negative effects from sticklebacks on piscivore populations can show large between area variation at relative small spatial scales.

To summarize, our results suggest that predation from three-spined stickleback can have strong negative effects on perch larval survival in areas where sticklebacks and larval stages of perch are not spatially or temporally separated. Hence, in areas where stickleback density is high, variation in effects on piscivore populations can be expected at relatively small spatial scales, depending on spawning site-specific characteristics ranging from coastal lakes and wetlands with strong migration barriers for sticklebacks to open bays with high accessibility.

### Reciprocal intraguild predation, habitat separation, and alternative stable states in coastal ecosystems

Reciprocal or mutual intraguild predation interactions have been suggested to be an important mortality factor for juvenile predators at high consumer densities (Fauchald 2010; Irigoien and de Roos 2011). Based on either the juvenile competition bottleneck hypothesis or reciprocal intraguild predation hypothesis, Walters and Kitchell (2001) suggested the presence of two alternative stable community states, a high predator density state where predators control the prey population, or a high prey population density state where prey limit predator population growth and density, i.e., the cultivation depensation hypothesis (see also Post et al. 2002). This has been suggested to be one possible mechanism behind the apparent lack of recovery of overfished piscivore populations (Post et al. 2002; Casini et al. 2009, but see De Roos and Persson 2002; Persson et al. 2007b for other mechanisms). In spatially linked ecosystems, the likelihood for ASS has been shown to depend on the degree of habitat separation (van der Hammen et al. 2010) or on the relative productivity of different habitats (Schreiber and Rudolf 2008). The three-spined stickleback/coastal piscivore intraguild predation system involves an extensive time period with spatial separation between coastal predators and sticklebacks due to the offshore life stage of the latter. These characteristics suggest a substantial decoupling of predation and energy gain of piscivores and stickleback abundance. Instead, piscivore population densities will to a large extent be dependent on coastal resource dynamics. Such intraguild predation scenarios suggest that the strength of top–down control of stickleback populations from coastal piscivores depends on the environmental conditions and resource production at the coast for the piscivore populations (van Leeuwen et al. 2013). Therefore, an increase in stickleback density can occur independently of coastal piscivore densities due to other factors like decreased predation pressure during the offshore stage (Casini et al. 2009; Eriksson et al. 2011) or increased productivity and food availability in offshore habitats (Lefebure et al. 2014, Olsson et al. unpubl.). In this scenario, increased densities of sticklebacks may have no or only minor positive effects on individual piscivore growth. Effects on coastal piscivore densities are instead likely to be negative via increased predation on the early stages of piscivores followed by reduced piscivore recruitment. Once the negative effects on recruitment of coastal predators occur, a feedback loop is created, with a decreasing predator control of coastal stages of sticklebacks. This will cause further increases in stickleback densities, leading to a coastal ecosystem state dominated by stickleback and with low densities of perch and pike.

From a management perspective, our results suggest that geographically constrained coastal fish communities in combination with reciprocal intraguild predation interactions and cross-ecosystem migrations between offshore and costal habitats are especially challenging. This is mainly due to two factors. First of all, the persistence of coastal piscivore populations is dependent on the availability of recruitment habitats where negative interactions with temporarily high densities of offshore planktivores can be avoided. Secondly, positive responses of coastal piscivore populations to increasing densities of offshore planktivores and hence increased top–down control of the latter cannot be expected.

## Electronic supplementary material

Supplementary material 1 (PDF 161 kb)
