# Supplementary material for: Declining coastal piscivore populations in the Baltic Sea: Where and when do sticklebacks matter?
Source: Ambio. 2015 May 28;44(Suppl 3):462–71. doi: 10.1007/s13280-015-0665-5 (PMC4447698; doi:10.1007/s13280-015-0665-5)
Supplement: Supplementary file 1 — Supplementary material 1 (PDF 161 kb) [file 13280_2015_665_MOESM1_ESM.pdf]

AMBIO

Electronic Supplementary Material
This supplementary material has not been copy edited by the publisher or the editorial office.

Title: **Declining coastal piscivore populations in the baltic sea: Where and when do sticklebacks matter?**

Authors: Pär Byström, Ulf Bergström, Alexander Hjälten, Sofie Ståhl, David Jonsson and Jens Olsson

Supplementary Material S1

MATERIALS AND METHODS

Laboratory experiment and gape size limitation

In order to estimate gape limitations of sticklebacks when feeding on differently sized perch larvae, we measured stickleback gape sizes and conducted a feeding experiment using differently size combinations of sticklebacks as predators and young-of-the-year (YOY) perch as prey. Sticklebacks were collected in June 2011 with a beach seine or hand net at the coastal bays Laxögern and Yttre Spelgrundet (Table S1, Fig. 1). Thereafter the fish were kept in a large holding tank (600 L) in a laboratory environment and fed with a mixture of live zooplankton, macroinvertebrates and perch larvae. Gape size of a random sample of sticklebacks was measured to the nearest 0.1 mm as the distance between the upper and lower jaw at a gape angle of 90°, using a stereo microscope (size range 26-78 mm, (n = 103). To estimate theoretical maximum length of a perch that differently sized sticklebacks can consume we combined our estimate of stickleback gape size relationship with previously derived relationships between YOY perch body height and length (Byström et al. 2012). Sticklebacks were sorted into three size classes (n = 8 in each class): small (30-40 mm), medium (45-55 mm) and large (65-75 mm), and placed individually in aquarium (water volume 25 L) with a water temperature of 19-20°C. Each aquarium contained a bottom positioned stone (Ø ≈ 5 cm), had continuous oxygen supply, and 1/3 of the water volume was exchanged weekly. Between the feeding experiments, sticklebacks were fed a mixture of live zooplankton, macroinvertebrates and perch larvae every second day. With signs of decreased feeding activity individual sticklebacks were replaced with new ones. As prey for the sticklebacks in the experiment we used six size classes of YOY perch (11, 14, 19, 21, 25 and 31 mm TL) obtained from egg strands collected in middle of May at the coastal bay Yttre Spelgrundet (Table S1). Perch was hatched and raised in large holding tanks (600 l, water temperature 18-19°C) in the laboratory, and were fed live zooplankton from a nearby pond. Prior to experiments, sticklebacks were starved for 24 hours. The stone at the bottom of the aquaria were removed and after being size sorted and measured to nearest 1 mm, either ten YOY perch (size classes 11 and 14 mm) or five YOY perch (size classes 19 to 31 mm) were introduced to each aquarium. Number of consumed perch was recorded after 4 hours and the remaining perch removed.

Pond experiment

We conducted a large-scale pond experiment to examine whether or not survival of YOY perch is dependent on their size when sticklebacks migrate into perch spawning sites. The experiment was conducted in two similar size ponds (32 × 10.8 m) situated four meters apart, close to Umeå University (Fig. 1). Both ponds have a well-developed *Carex sp.* belt along the shoreline and scattered submerged vegetation (*Potamogeton sp.*) cover the bottom substrate. As such, the ponds contain natural prey communities for juvenile fish holding both zooplankton and macroinverteberates (e.g. Byström and Andersson 2005). Each pond was further divided into eight enclosures (size 4 × 10.8 m, mean depth 0.90 m) with a reinforced dark green plastic sheet making in total 16 enclosures. Each enclosure had an inflow of water of 0.1 m^3/h and as each pond only had one outlet, each wall (sheet) had an opening (width 0.2m) that reached from the bottom to the water surface covered with a metal net (mesh size 1 mm) to allow for water flow to the outlet. We used a design with four treatments with temporal variation in stickleback presence and one control replicated 3 times each, making in total 15 enclosures. In addition, the last enclosure was treated as the controls but was sampled for size distribution of perch larvae each time when sticklebacks were introduced to a treatment enclosure. Perch larvae originated from four egg strands collected from Yttre Spelgrundet (Table S1, Fig. 1) and brought back and hatched in a 600 liter holding tank at Umeå University. Similarly, adult sticklebacks were captured with a hand net at Yttre Spelgrundet on 26th May and kept in a 600 l holding tank at Umeå University. Perch larvae were fed daily with live zooplankton collected from a nearby pond and adult sticklebacks a mixture of live zooplankton, macroinvertebrates and frozen chironomids prior to introduction into the enclosures.

300 (6.9 individuals m^{-2}) first feeding perch larvae (7.2 ± 0.26 mm, mean ± 1 SD) were introduced at 28th of May into each of the 16 enclosures. Thereafter, six (0.14 individual's m^{-2}) adult sticklebacks (62.5 ± 4.7 mm, mean ± 1 SD) were introduced either 1, 8, 17 or 24 days after the introduction of perch larvae. The stickleback density chosen is low compared to natural densities where up to 30 individuals m^{-2} could be found in some coastal areas (Eriksson et al. 2011). Perch were sampled with a large hand net at day 8, 17, and 24 in the enclosure used only for control perch size development (body length, mean ± 1 SD = 9.0 ± 0.3, 11.5 ± 0.8, and 15.4 ± 2.0 mm on respectively sampling day, n = 10 - 20). In each treatment perch and sticklebacks were sampled 18-19 days after introduction of sticklebacks, i.e. day 19, 27, 35 and 43. Fish were sampled in each enclosure with a fine mesh seine net, and for each enclosure sampling was terminated when two subsequent hauls rendered zero catches of YOY perch.

Sampled perch were conserved in Lugol’s solution and later in the laboratory measured to the nearest 0.1 mm (total length).

Zooplankton densities was sampled with a 100 µm-mesh net (diameter 250 mm) drawn 3.5 m horizontally at a depth of 0.1m in the deepest part of the enclosures at the introduction of perch larvae and at the day prior to termination of each treatment. Zooplankton was preserved in Lugol´s solution. In the laboratory, zooplankton individuals were counted and classified to suborder, family or genus, and the body lengths of 15 individuals (all if fewer), of each category from each sample were measured. Lengths were transformed to biomass using regressions relating length to dry weight (Dumont et al. 1975; Botrell et al. 1976).

Field studies

Densities of perch larvae at coastal spawning sites

In order to study if there is any relationship between changes over time in perch larvae density and stickleback density, we sampled perch spawning sites along the Bothnian Sea coast in the years 2011 and 2012 (Fig. 1, Table S1). The sites were selected based on mapping of perch recruitment areas made by the regional County board authorities in 2011 (Länsstyrelsen Västerbotten 2013; Länsstyrelsen Västernorrland 2013). Larval perch were sampled approximately weekly, or every second week, approximately from hatching (Table 1) to an age of five weeks with a bongo-trawl (∅ 0.60 m, length 4 m, mesh size of 5 mm). The trawl was held by a steel construction at the prow 0.6 m out on one side of the boat, for further details on equipment see Byström et al. (1998). Trawling was made during day-time between 10.00 and 15.00, and at least four stations were sampled in each bay and date. In all but one bay the trawling depth was 0.1 m, whereas in Häggvik, trawling was done at two depths (0.1m and 1.6 m) as this bay was substantially deeper than the others (Table S1). If very few or no larvae were caught at a site, sampling was cancelled. We defined newly hatched perch larvae as perch smaller than 6.5 mm which approximately include larvae hatched up to a week earlier (c.f. Wang and Eckman 1994). All captured larvae were counted and individual subsamples were preserved in Lugol´s solution for later length measurement in the laboratory (total length, to the nearest 0.1 mm).

Concomitant with the trawling, 16 to 22 Ella traps (www.ellafishing.com) (∅ 0.70 m, height 0.6 m, mesh size 6 mm) were set over night (set 15.00 -17.00 and collected the following morning at 08.00 -10.00) approximately 10 m apart along the shore line at a depth of 1 – 2 m to obtain a

relative measure of stickleback abundance in each bay. The sticklebacks were counted and thereafter released back to the bay after a subsample of sticklebacks was collected and frozen for later diet analyses in the laboratory. To assess the zooplankton abundance in the different bays, three pelagic stations in each bay were sampled for zooplankton using a 100 µm-mesh net (diameter 250 mm) either drawn three meters horizontally at a depth of 0.3 meters from the water surface (2011), or drawn vertically from 1 meters depth to the surface six times per station (2012). Samples were preserved in Lugol´s solution, and zooplankton taxa were counted and biomass obtained according to the same method as applied in the pond study (see above). In this study we report data on YOY perch abundances and stickleback catches from only two of the sampling sites, Yttre Spelgrundet and Västra Stadsviken, which were sampled in both 2011 and 2012 as the complete data set is used in another article (Byström and Wennhage, unpubl.). However, in this study we use the whole dataset to study variation in stickleback caches at the spawning sites over perch ontogeny.

Case study

Results from coastal survey gillnet monitoring programs show that stickleback abundance in the large bay Gaviksfjärden (both Häggvik and Sörleviken is sub bays in Gaviksfjärden) has increased substantially from year 2004 to 2012 (Appelberg et al. 2013, Lingman 2013). Mean captures in shallow areas (0-6 m) have varied between 123 - 316 individuals per net between years in 2010 – 2012, which is substantially higher than catches in any other monitoring site along the Bothnian Sea coast (Olsson et al.unpublished). Moreover, total trap catches (22 traps) in Sörleviken (Table S1), on a spawning site for perch in Gaviksfjärden, amounted to 8650 adult sticklebacks in early June along a 400 m shore-line (c.f. Table 1, results). Still, the suggested negative effects of sticklebacks on perch populations have not been observed in Gaviksfjärden despite high densities of sticklebacks (Appelberg et al 2013, Lingman 2013). In Gaviksfjärden there are at least two freshwater outlets, one in Häggvik and one in Sörleviken, that connects the coast with closely situated (ca 400 meters) freshwater lakes. According to local fishermen, perch migrate to these lakes for spawning in spring.

To investigate the importance of freshwater lakes as spawning sites for perch in Gaviksfjärden, we estimated the number of upstream migrating perch in spring 2013 to the small shallow lake (3 ha, max depth 1.8m) 400 m upstream Sörleviken using a fish counter (Vaki river fish counter) set approximately 150 m upstream the outlet to Sörleviken. The fish counter uses infrared scanning technology to count and identify species and sizes of fish that passes the

scanners (for details, see www.vaki.is/Products/RiverwatcherFishCounter). Recording of fish migration started on 2nd of May and here we present recordings of perch upstream migration until the hatching of perch larvae in the lake at 29th of May. Bongo trawling to estimate YOY perch abundance was carried out in 2013 in the connected lake to study densities of perch larvae in the lake. Three stations were sampled during two occasions (29th of May and 6th of June) as the growth of high-density belts of macrophytes prevented efficient trawling at later dates. Six Ella traps were set over night on the 29th of May and 6th of June in the lake close to the outlet to assess whether or not stickleback migrated up to the lake or were present in the lake. In addition to this, the contribution to the resident perch population in Gaviksfjärden of freshwater recruited perch was assessed using otolith micro-chemistry analysis of strontium (Sr) and calcium (Ca) concentrations of 28 adult perch (three years old), captured in August 2010 using coastal multimesh gillnets (Lingman 2013). The concentrations of Sr and Ca at points every 6th micrometer along a transect from the inner part of the otolith (birth) to the edge (present), was analyzed using proton induced x-ray emission at the Nuclear Microprobe Laboratory at the Department of Physics, Lund University, Sweden. For further details on preparation of otoliths, reference fish and analysis see Engstedt et al. (2012) and Wastie (2013).

REFERENCES

Appelberg M., M. Bergenius, U. Bergström, M. Casini, A. Gårdmark , J. Hjelm, M. Huss, O. Kaljuste, J. Olsson, U. Sahlin, H. Wennhage, and M. Werner. 2013. PLAN FISH: Planktivore management linking food web dynamics to fisheries in the Baltic Sea: slutrapport för Skarpsillsprojektet 008-2013. Department of Aquatic Resources, SLU.

Bottrell, H. H., A. Duncan, Z. M. Gliwicz, E. Grygierek, A. Herzig, A. Hillbrichtilkowska, H. Kurasawa, P. Larsson, and T. Weglenska. 1976. Review of some problems in zooplankton production studies. Norwegian Journal of Zoology 24:419-456.

Byström, P., and J. Andersson. 2005. Size-dependent foraging capacities and intercohort competition in an ontogenetic omnivore (Arctic char). OIKOS 110:523-536.

Byström, P., L. Persson, and E. Wahlström. 1998. Competing predators and prey: juvenile bottlenecks in whole-lake experiments. Ecology 79:2153-2167.

Byström, P., M. Huss, and L. Persson. 2012. Ontogenetic constraints and diet shifts in Perch (*Perca fluviatilis*): mechanisms and consequences for intra-cohort cannibalism. Freshwater Biology 57:847-857.

Dumont, H. J., I. Vandevelde, and S. Dumont. 1975. Dry weight estimate of biomass in a selection of cladocera, copepoda and rotifera from plankton, periphyton and benthos of continental waters. Oecologia 19:75-97.

Engstedt, O., P. Stenroth, P. Larsson, L. Ljunggren, and M. Elfman. 2010. Assessment of natal origin of pike (*Esox lucius*) in the Baltic Sea using Sr:Ca in otoliths. Environmental Biology of Fishes 89:547-555.

Eriksson, B. K., K. Sieben, J. Eklöf, L. Ljunggren, J. Olsson, M. Casini, and U. Bergström. 2011. Effects of altered offshore food webs on coastal ecosystems emphasize the need for cross-ecosystem management. Ambio 40:786-797.

Lingman, A. (2013). Faktablad från regional kustövervakning i Bottniska viken, Gaviksfjärden 2004-2012.

Länsstyrelsen Västerbotten. 2013. Fiskrekrytering efter Västerbottenskusten. Meddelande 2. In Swedish.

Länsstyrelsen Västernorrland. 2013. Lek och uppväxtområden för fisk längs Västernorrlands kust. Slutrapport Lst, dnr. 511-6745-12. In Swedish.

Wang, N. and R. Eckmann. 1994. Effects of temperature and food density on egg development, larval survival and growth of perch (*Perca fluviatilis* L.). Aquaculture 122:323-333.

Wastie J. 2013. Assessing the importance of freshwater tributary systems for the recruitment of Eurasian perch (*Perca fluviatilis*) in Baltic Sea coastal ecosystems. Master thesis report. Department of Aquatic Resources, Swedish University of Agricultural Sciences, Sweden.

.

Table S1 Coordinates, estimated maximum depth and temperature at hatching of perch larvae of sampled spawning sites. When two temperatures are given this represents year 2011 and 2012, respectively. * No larvae found and hatching date assumed to be the same as in Häggvik. n.a. data not available.

Site	Coordinates (lat-lon)		Max depth (m)	Temperature at hatching (°C)
A) Laxögern	63°45'29" N	20°33'16" E	1.5	12.1
B) Inre Spelgrundet	63°45'16" N	20°32'44" E	2.1	13.2
C) Yttre Spelgrundet	63°45'14" N	20°32'18" E	1.7	12.1 : 13.3
D) Boviken	63°36'49" N	19°59'50" E	1.4	n.a.
E) Inre Stadsviken	63°33'21" N	19°47'55" E	1.5	12.7
F) Yttre Stadsviken	63°33'22" N	19°47'41" E	1.5	10.7 : 11.4
G) Tennavan	63°28'80" N	19°24'90" E	2.6	11.9
H) Inneravan	63°28'47" N	19°19'50" E	2.0	12
I) Sörleviken (Gaviksfjärden)	62°53'00" N	18°20'18" E	3.5	12.7*
J) Häggvik (Gaviksfjärden)	62°54'33" N	18°17'18" E	> 6	11.9
K) Coastal lake, Sörleviken (Gaviksfjärden)	62°53'26" N	18°20'43" E	1.7	16.7 (2013)

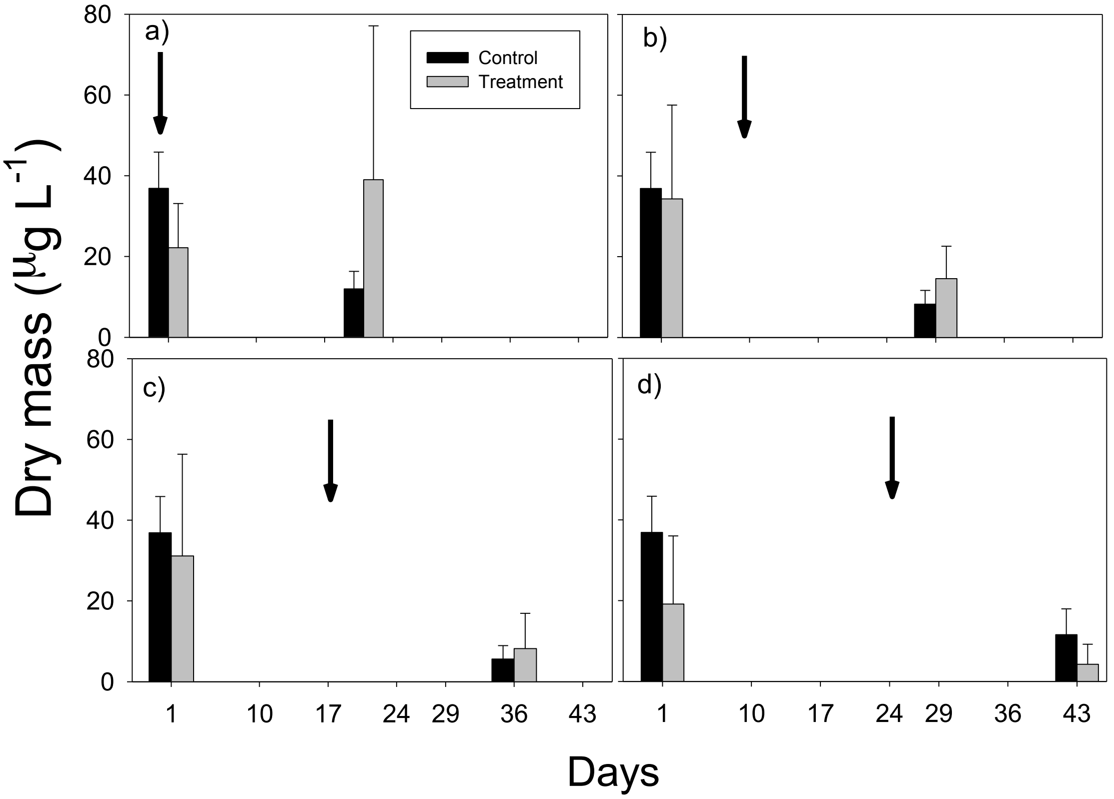

Fig. S1 Zooplankton biomass in treatment and control enclosures at start and termination of each treatment. (a) stickleback introduction day 1, (b) day 8, (c) day 17a and (d) day 24, respectively. Arrows denote time when sticklebacks were introduced in each treatment.
